# Supplementary material for: Strong ice-ocean interaction beneath Shirase Glacier Tongue in East Antarctica
Source: Nat Commun. 2020 Aug 24;11:4221. doi: 10.1038/s41467-020-17527-4 (PMC7445286; doi:10.1038/s41467-020-17527-4)
Supplement: Supplementary file 1 — Supplementary Information [file 41467_2020_17527_MOESM1_ESM.pdf]

Supplementary Information for
"Strong ice-ocean interaction beneath Shirase Glacier Tongue in East Antarctica"

Hirano et al.

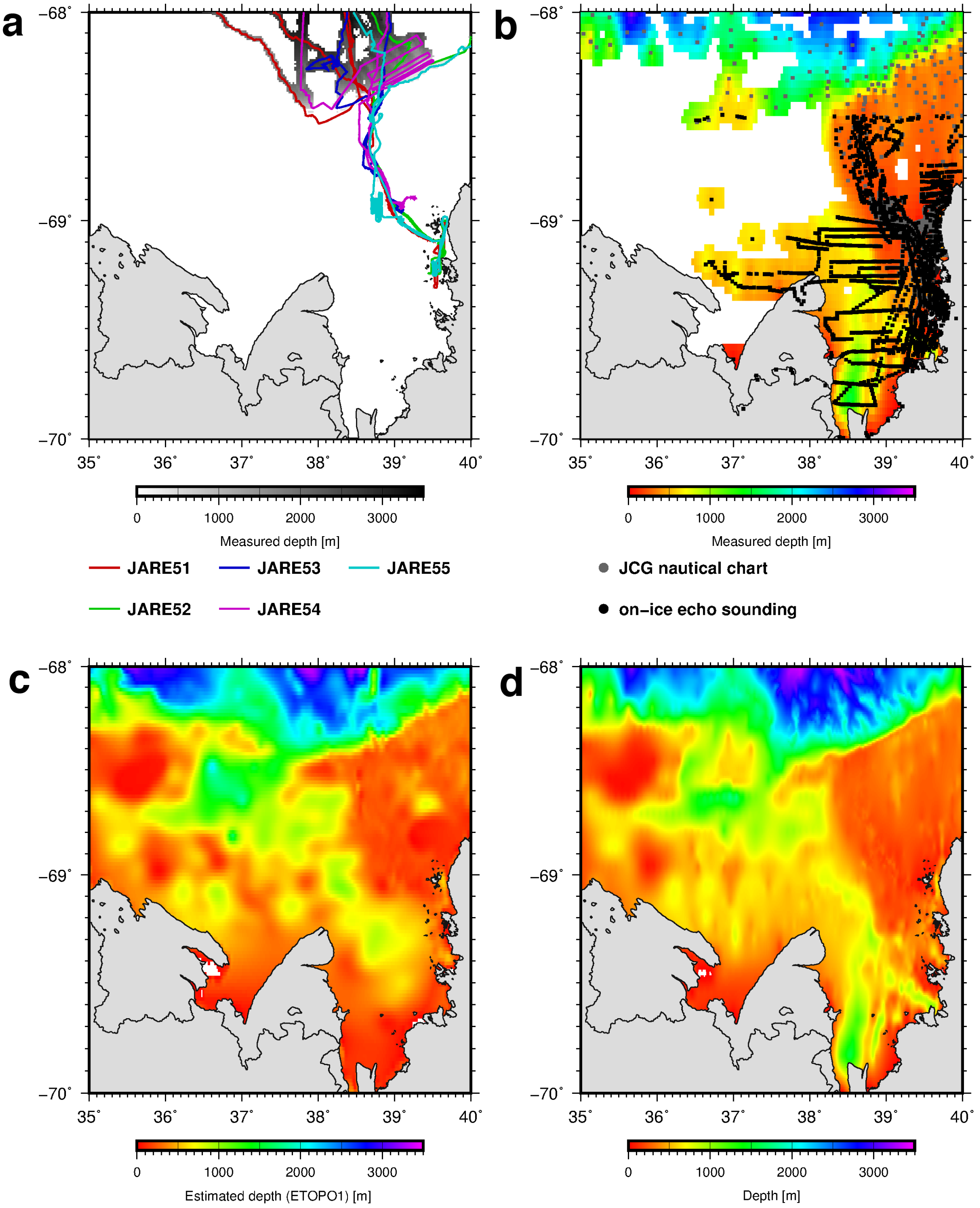

Supplementary Figure 1 | Bathymetric map of (a) multibeam echo sounder data, (b) on-ice echo sounding data (black circles) and hydrographic survey-based nautical chart data (gray circles), (c) ETOPO1[1], and (d) compiled 1-km gridded digital terrain model utilized for physical oceanographic modeling. Ship tracks of the Japanese Antarctic Research Expedition (JARE) 51st (red), 52nd (green), 53rd (blue), 54th (purple), and 55th (sky blue) for multibeam bathymetry are shown in (a).

Supplementary Note 1 | Past hydrographic data in Lützow-Holm Bay during the 31st and 32nd Japanese Antarctic Research Expedition in 1990-1992 (On-ice observations)

During the wintering periods of the 31st and 32nd Japanese Antarctic Research Expedition (JARE 31st and JARE 32nd) in 1990-1992, hydrographic observations were carried out at three trough stations in Lützow-Holm Bay (Sta.OW4, L4, and P2, Supplementary Fig. 2) using a conductivity-temperature-depth profiler (CTD; Sea-Bird Electronics SBE19), as a part of Antarctic Climate Research Program[2]. A snow vehicle was used to access the observation sites on the land-fast sea ice, and CTD observations were conducted using holes drilled thorough the fast ice (namely, on-ice observations), because for both wintering periods the bay was entirely covered with heavy land-fast sea ice. The dates of the observations for the three CTD stations were: 30 August and 29 October 1990 at Sta.OW4, 6 May, 24 August, and 23 October 1990 at Sta.L4, and 27 April, 18 August, 17 October 1990, 27 January 1991, and 18 January 1992 at Sta.P2. In addition, we use time-series data of subsurface temperature and current velocity at Sta.P2 (also shown as a yellow star in Fig. 1a) from late January to August in 1990, obtained using a mooring equipped with a single current profiler (Aanderaa current meter RCM-7) suspended from the fast ice at 300 dbar (see Figs. 6a and 6b).

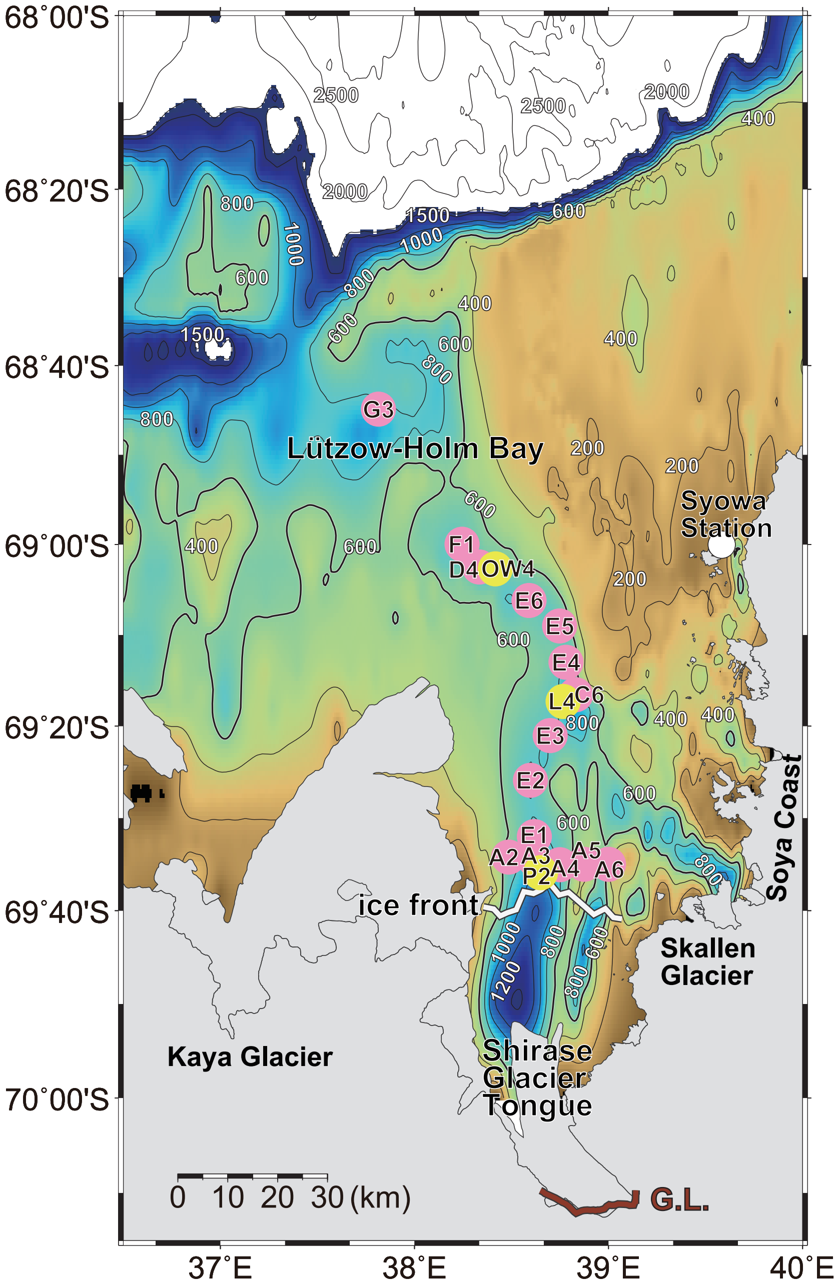

Supplementary Figure 2 | Locations of on-ice CTD observations conducted for two wintering periods in 1990-1992 (yellow circles, Sta.OW4, L4, and P2 in the deep trough). A subsurface mooring was deployed at Sta.P2 (also shown by yellow star in Fig. 1a), with a current profiler located at 300 dbar. The mooring ran from late January to August in 1990 (see Figs. 6a and 6b). For reference, the positions of shipboard CTD observations in 2017 (pink circles) are also shown (only for the stations used in Fig. 2). Thick white line shows the SGT ice front derived from MODIS imagery on 22 January 2017 (see Fig. 1a). Thick brown line represents the SGT grounding line[3].

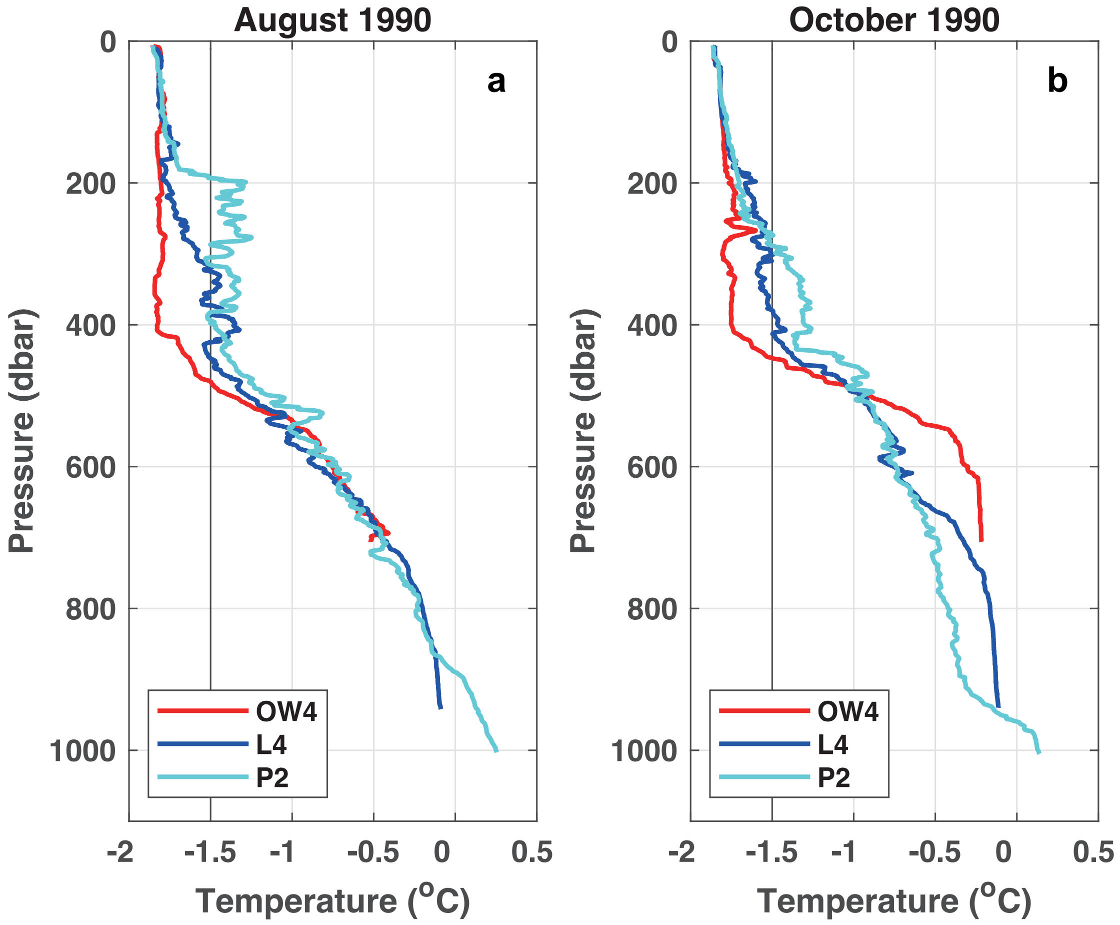

Supplementary Figure 3 | Vertical profiles of temperature at three trough stations (Sta.OW4, L4, and P2) in (a) August and (b) October 1990. For emphasis, T = -1.5°C, defined as the lower boundary of Winter Water layer[4], is denoted by vertical thick line.

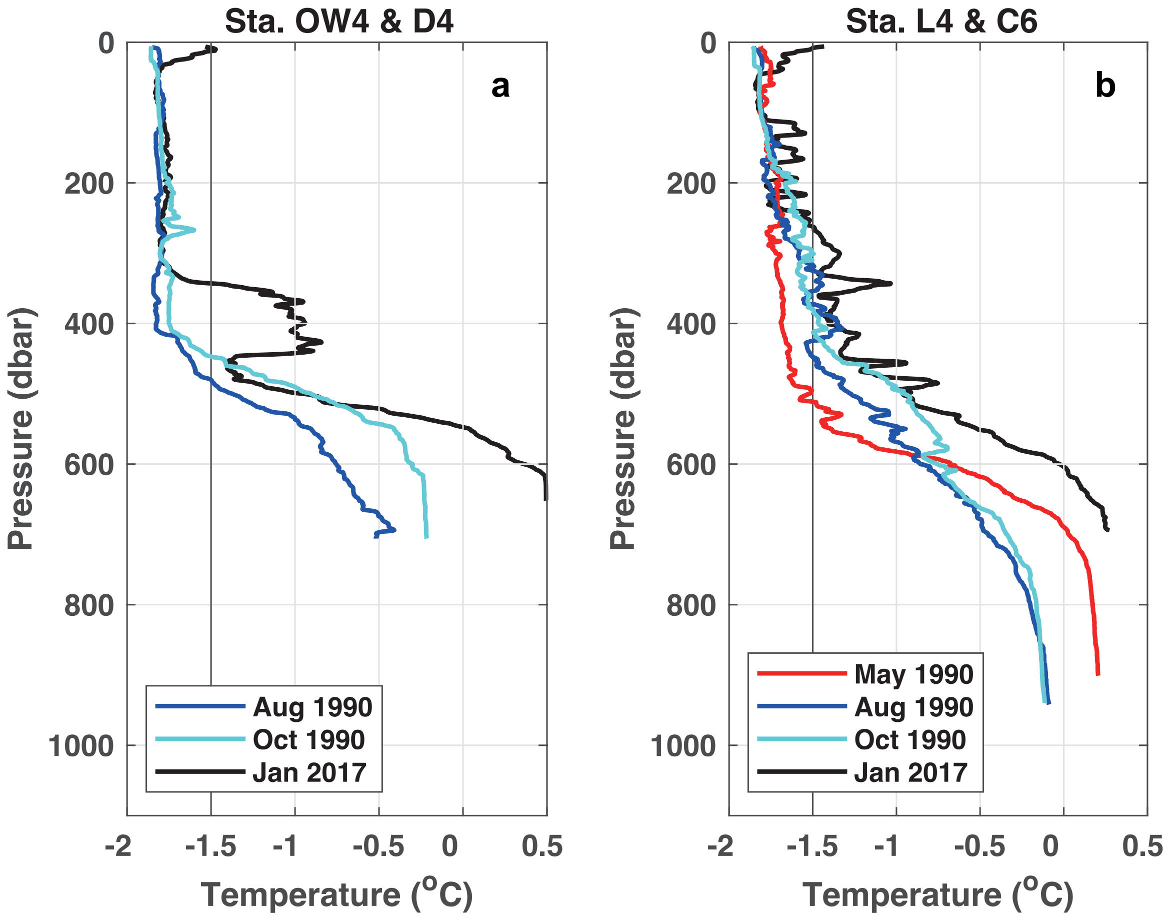

Supplementary Figure 4 | Vertical profiles of temperature obtained in May, August, and October 1990 (JARE 31st and JARE 32nd), and January 2017 (JARE 58th) at two trough stations for (a) Sta.OW4 (color) and D6 (black) and (b) Sta.L4 (color) and C6 (black). Note that positions of Sta.D4 and C6 (JARE 58th) are almost the same as those of Sta.OW4 and L4 (JARE 31st and 32nd), respectively (see Supplementary Fig. 2). For emphasis, T = -1.5°C, defined as the lower boundary of Winter Water layer[4], is denoted by vertical thick line.

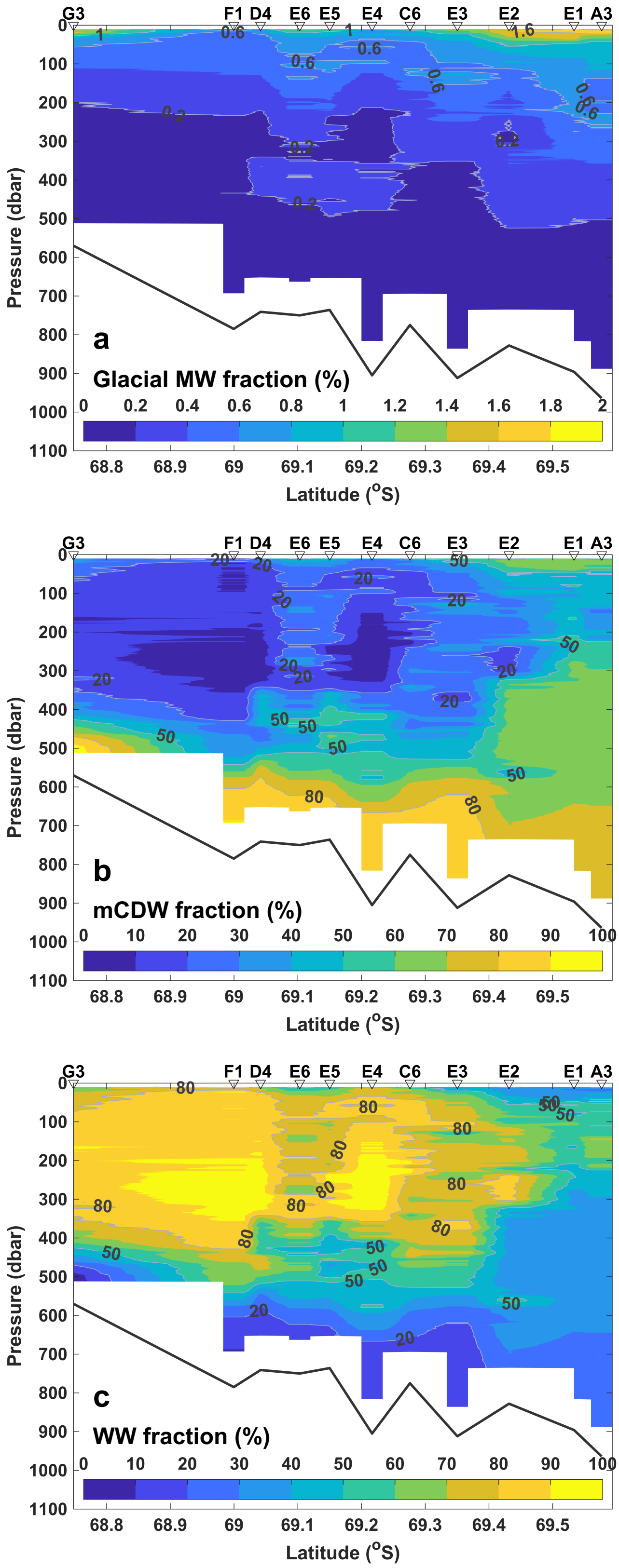

Supplementary Figure 5 | Along-trough vertical sections of (a) glacial meltwater, (b) mCDW, and (c) WW fractions (%) estimated from CTD-measured profiles (see Methods). Stations used in panels (a)-(c) are the same as those used in Figs. 2a-2c.

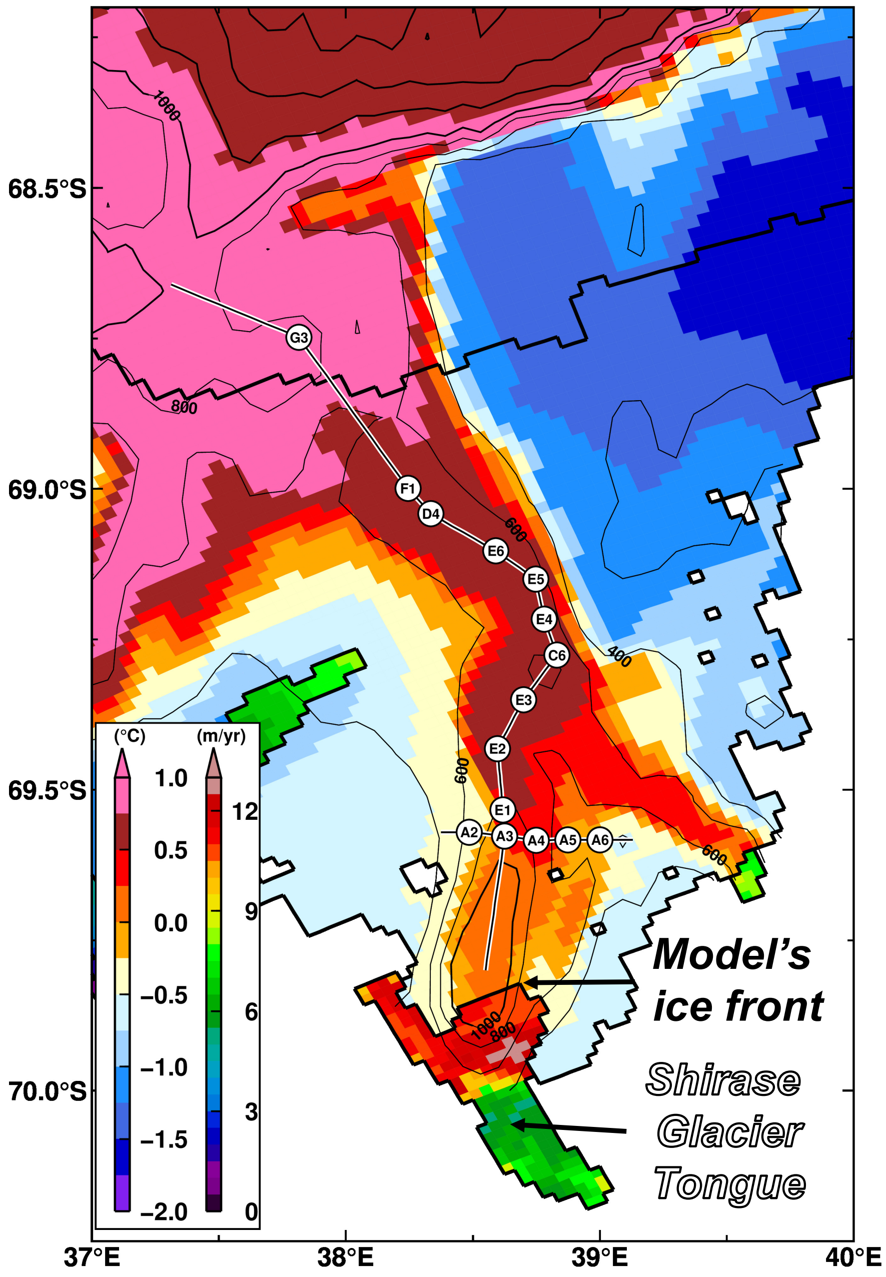

Supplementary Figure 6 | Simulated climatology of bottom temperature (°C) in Lützow-Holm Bay and annual basal melt rate of Shirase Glacier Tongue (m yr^{-1}) in January (averaged for 2006-2017, results from NOFI case). Note that model's ice front is located south of the observed ice front derived from MODIS imagery on 22 January 2017 (see Fig. 1a).

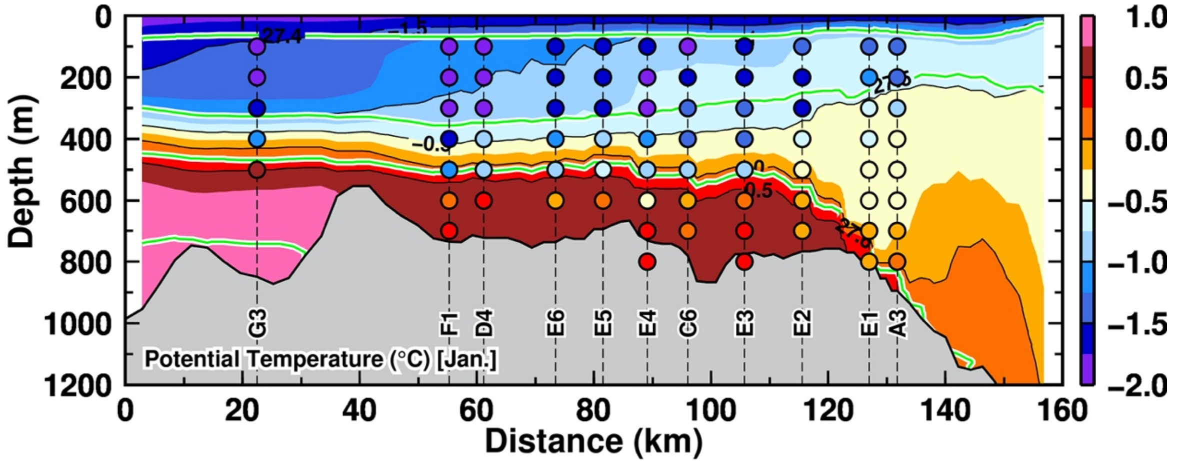

Supplementary Figure 7 | Along-trough vertical section of simulated climatology of temperature (°C) in January (averaged for 2006-2017, results from NOFI case). Each color of dot denotes the observed temperature in January 2017 (see also Fig. 2a).

Supplementary References:

1. Amante C, Eakins BW. ETOPO1 1 Arc-Minute Global Relief Model: Procedures, Data Sources and Analysis. *NOAA Technical Memorandum* **NESDIS NGDC-24**, 19 (2009).

2. Takizawa T, Ushio S, Kawamura T, Ohshima KI, Ono N, Kawaguchi S. Preliminary results of hydrography under fast ice in Lutzow-Holm Bay, Antarctica in 1990. *Proc NIPR Symp Polar Meteorol Glacial* **6**, 106-125 (1992).

3. Yamanokuchi T, Doi K, Shibata Y. Validation of grounding line of the East Antarctic Ice Sheet derived by ERS-1/2 interferometric SAR data. *Polar Geoscience* **18**, 1-14 (2005).

4. Ohshima KI, Takizawa T, Ushio S, Kawamura T. Seasonal variations of the Antarctic coastal ocean in the vicinity of Lutzow-Holm bay. *J Geophys Res-Oceans* **101**, 20617-20628 (1996).
